# A Social Media–Based Intervention for Chinese American Caregivers of Persons With Dementia: Protocol Development

**DOI:** 10.2196/40171

**Published:** 2022-09-29

**Authors:** Y Alicia Hong, Kang Shen, Huixing Kate Lu, Hsiaoyin Chen, Yang Gong, Van Ta Park, Hae-Ra Han

**Affiliations:** 1 Department of Health Administration and Policy College of Public Health George Mason University Fairfax, VA United States; 2 Chinese Culture and Community Service Center, Inc Gaithersburg, MD United States; 3 School of Biomedical Informatics University of Texas Health Science Center at Houston Houston, TX United States; 4 Department of Community Health Systems School of Nursing University of California, San Francisco San Francisco, CA United States; 5 School of Nursing Johns Hopkins University Baltimore, MD United States

**Keywords:** Alzheimer disease, dementia, caregivers, Chinese Americans, mHealth intervention, mobile health, WeChat, social media, aging

## Abstract

**Background:**

Racial/ethnic minority and immigrant caregivers of persons with dementia experience high rates of psychosocial stress and adverse health outcomes. Few culturally tailored mobile health (mHealth) programs were designed for these vulnerable populations.

**Objective:**

This study reports the development of a culturally tailored mHealth program called Wellness Enhancement for Caregivers (WECARE) to improve caregiving skills, reduce distress, and improve the psychosocial well-being of Chinese American family caregivers of persons with dementia.

**Methods:**

Community-based user-centered design principles were applied in the program development. First, the structure and curriculum of the WECARE program were crafted based on existing evidence-based interventions for caregivers with input from 4 experts. Second, through working closely with 8 stakeholders, we culturally adapted evidence-based programs into multimedia program components. Lastly, 5 target users tested the initial WECARE program; their experience and feedback were used to further refine the program.

**Results:**

The resulting WECARE is a 7-week mHealth program delivered via WeChat, a social media app highly popular in Chinese Americans. By subscribing to the official WECARE account, users can receive 6 interactive multimedia articles pushed to their WeChat accounts each week for 7 weeks. The 7 major themes include (1) facts of dementia and caregiving; (2) the enhancement of caregiving skills; (3) effective communication with health care providers, care partners, and family members; (4) problem-solving skills for caregiving stress management; (5) stress reduction and depression prevention; (6) the practice of self-care and health behaviors; and (7) social support and available resources. Users also have the option of joining group chats for peer support. The WECARE program also includes a back-end database that manages intervention delivery and tracks user engagement.

**Conclusions:**

The WECARE program represents one of the first culturally tailored social media–based interventions for Chinese American caregivers of persons with dementia. It demonstrates the use of community-based user-centered design principles in developing an mHealth intervention program in underserved communities. We call for more cultural adaptation and development of mHealth interventions for immigrant and racial/ethnic minority caregivers of persons with dementia.

## Introduction

Currently, more than 6 million Americans aged ≥65 years are living with Alzheimer disease or related dementias (ADRD) [1]. More than 11 million family caregivers of persons with dementia provide an estimated 15.3 billion hours of unpaid care valued at US $255.7 billion a year [1]. Family caregivers of people with dementia have high rates of emotional distress and negative health outcomes [2]. For example, 59% of caregivers of persons with dementia reported high rates of emotional stress [3], 40% reported depression, and 44% reported anxiety [4-6]. Family caregivers of persons with dementia also reported higher levels of physical stress [3,7], lower quality of sleep [8-10], and lower quality of life [11-13]. Some caregivers developed chronic conditions including impaired immune functions, hypertension, and coronary health diseases [14]. As the US population is aging and the number of persons with dementia is expected to reach 13 million by 2050, the burden on family caregivers and their psychosocial well-being requires more public health attention [1].

Asian Americans represent the fastest-growing racial group in the United States; they accounted for 7% of the total US population in 2020 and are projected to reach 12% in 2050. Chinese Americans represent nearly a quarter (23%) of the Asian American population [15]. The literature on Chinese American caregivers of persons with dementia is limited and mostly descriptive. Available literature indicates that cultural values of family harmony and the practice of filial piety permeate all aspects of the Chinese American caregiving process, including their appraisal of stress and coping strategies [16]. Caring for older family members is not only a sign of love and pride but also a moral obligation [17,18]. Compared to their White counterparts, Chinese Americans are more likely to live in multigenerational households [19]. Chinese caregivers are often providers of young children and older adults while also being engaged in the formal labor force themselves [19,20]. Although caregiving can strengthen attachment and emotional bonds, it also leads to tensions and feelings of being neglected [18]. These frustrations are amplified in immigrant families where cultural differences often clash with generational gaps [20].

Most of dementia care is received at home where family caregivers play a central role but often lack the knowledge and skills to perform caregiving duties to meet the needs of persons with dementia. Chinese caregivers tend to keep problems within the family and do not seek external help because of the stigma associated with dementia or cognitive impairment [17]. The isolation and challenge are exacerbated by their minority and immigrant status, and those without English proficiency are further marginalized [21]. Most Chinese caregivers have limited knowledge and use of formal care and support services; they are also disconnected from “mainstream” dementia support groups due to language and cultural barriers [22]. Therefore, they endure higher levels of stress, mental disorders, and chronic conditions [23,24].

To date, few interventions have been conducted to serve the needs of Chinese American caregivers of persons with dementia. Specifically, in a recent systematic review, only 2 interventions targeting Chinese American caregivers were identified [25]. One was a home-based in-person behavioral management program [26], and the other was a DVD-based psychoeducation skills training program [27]. Both interventions, developed by Gallagher-Thompson and colleagues in early 2000s, cannot meet needs of a large population of Chinese persons with dementia, as face-to-face intervention is resource-intensive, and DVDs are not commonly present in most American households anymore. The need for accessible and scalable mobile health (mHealth) interventions has become more salient during the COVID-19 pandemic, when in-home visits or in-person services are prohibitive, and many people adopted telehealth or mHealth programs. However, the existing mHealth interventions for caregivers of persons with dementia were mainly designed for people of higher socioeconomic status with limited programs for racial/ethnic minority or immigrant populations. Adapting evidence-based interventions for digital delivery in underserved communities remains a critical literature gap. As racial/ethnic minority and low-income populations are more likely to be smartphone-dependent for internet access (without a computer or other mobile devices) and rely on social media as a primary source of health information [28], an mHealth intervention delivered via a social media app used by the target population is a logic solution. Nevertheless, evidence or research in this regard is rather limited. For example, in a recent systematic review of mHealth interventions for dementia caregivers, none was delivered via a social media app [29].

Out of nearly 4 million Chinese Americans, more than 70% are foreign-born [30]. Over 3 million Americans speak Chinese, the third most spoken language, only after English and Spanish. As the Asian American population continues to grow, a culturally tailored and linguistically appropriate mHealth intervention has a tremendous potential to reach a large number of Chinese American caregivers of persons with dementia.

More than 95% of Chinese Americans own a smartphone [31]. Similar to other immigrants whose first language is not English, many Chinese Americans use a social media app of their native language. For example, WeChat, a highly popular social media app, has a 97% penetration rate in smartphone users in China and a 90% penetration rate in Chinese-speaking Chinese Americans [32]. As a social media app, WeChat has the functions of “moments” (sharing photos and stories with friends and receiving “likes” and feedback), texting, voice call, video call, private chat, group chat, location sharing, file transfer, and making/receiving payment. These built-in functions allow intervention developers to focus on the content, thus saving time and cost. It also enables easy adoption and long-term use, especially in low-income communities [33]. Thus, WeChat would be an efficient delivery channel for an mHealth intervention for Chinese Americans caregivers.

To address the literature gap and public health needs, our team developed a WeChat-based intervention called “Wellness Enhancement for Caregivers” (WECARE) for Chinese American family caregivers of persons with dementia. This paper reports the process of developing the WECARE program and the design features of this social media–based intervention.

## Methods

### Overview of Study Design

The study was conducted from September 2021 to April 2022 with the goal of developing a culturally tailored WeChat-based intervention to improve the psychosocial well-being of Chinese American family caregivers of persons with dementia. The development of the WECARE program consisted of 3 steps. First, with experts’ input, we designed the structure and curriculum of the WECARE program based on existing evidence-based behavioral interventions for persons with dementia. Second, using a community-engaged user-centered design, we developed the multimedia components of the WECARE program that used the built-in functions of the WeChat app; we also developed a back-end database to manage intervention delivery and track user engagement. Finally, the complete WECARE program was refined after a beta test in target users.

### Ethics Approval

The study protocol was approved by the Institutional Review Board of the George Mason University (IRB1849712). Informed consent was obtained from all participants prior to data collection. Given that this study was focused on protocol development, we did not collect participants’ personal information including their demographic data.

### Step 1: Structural Design Based on Experts’ Input and Evidence-Based Programs

We first conducted interviews with 4 experts in the fields of ADRD, caregiving, cultural adaption, and mHealth intervention development, with 1 expert from each field. We sought input from the experts on (1) how to apply existing theories and evidence to enhance caregiving skills and reduce psychosocial stress among underserved family caregivers, (2) how to adapt evidence-based caregiving interventions for Chinese American caregivers, and (3) how to identify and prepare for potential barriers and facilitators during the intervention delivery.

The experts also suggested that the curriculum design be based on evidence-based interventions proven to be effective in minority and underserved caregivers of persons with dementia. Specifically, we used the Resources for Enhancing Alzheimer’s Caregiver Health II for its major domains of the intervention [34], Building Better Care for its short courses and training materials [35], and the DVD program of Gallagher-Thompson and colleagues [27] for culturally relevant problems and solutions for Chinese American caregivers of persons with dementia [27].

### Step 2: Iterative Design With Key Stakeholders

Following the principles of community-engaged user-centered design [36] while developing the program components, we worked closely with 8 stakeholders, including Chinese American family caregivers of persons with dementia (n=3), health care providers (n=3), and community leaders (n=2). Weekly meetings were organized to seek immediate input from stakeholders in terms of cultural appropriateness, ease of use, user engagement, and error reduction. The program components underwent the iterative process of being developed, reviewed, revised, tested, and refined. We also hired a software engineer to develop a back-end database to manage the WECARE delivery and user profiles. The database reflects the required functions identified in Step 1, including prescheduled automatic intervention delivery, user profile management, and user engagement tracking.

### Step 3: Testing and Refinement

When the complete WECARE program was ready, we tested it among 5 target users. Of these 5 Chinese American family caregiver participants—1 man and 4 women—all had limited English proficiency and were recruited by our community partner. Participants were invited to a conference room and received the WECARE program on their WeChat accounts to test its navigation and the functions of the back-end database. During the test, participants were encouraged to “think out loud” and share their feedback while going through the program components. Participants were asked to check if all program components were delivered at prescheduled times and if all program components could be opened without problem. Meanwhile, research staff monitored all user activities on WECARE’s back-end database. All interviews were conducted in Chinese or Mandarin and audio recorded, and detailed notes were taken while observing users’ navigation behaviors. The research team discussed users’ feedback, addressed the glitches reported in the test, and further refined the program.

## Results

### Program Curriculum and Components

**Figure 1:**
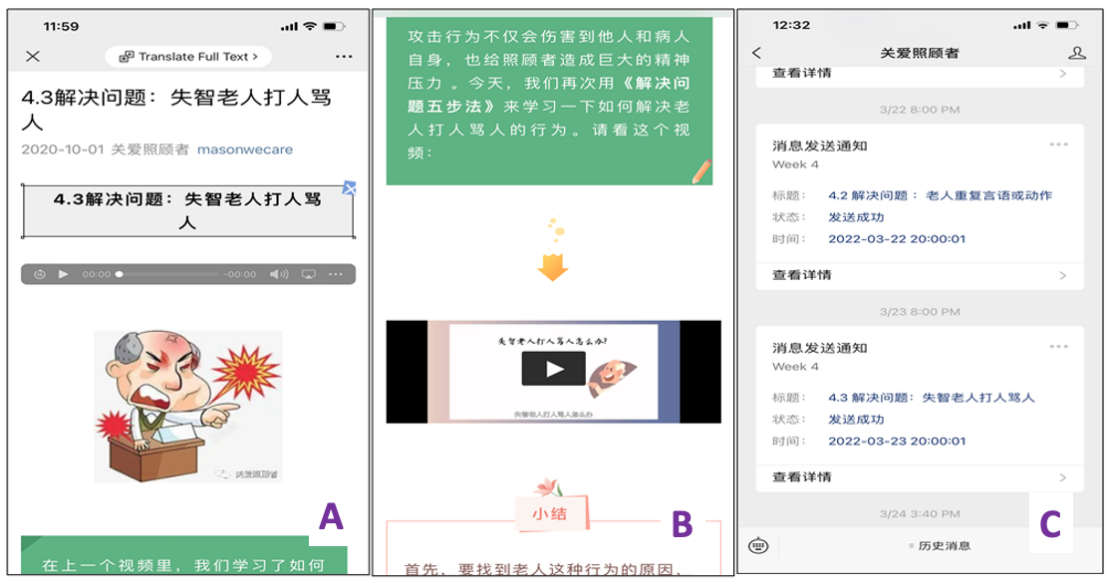
Wellness Enhancement for Caregivers (WECARE) screenshots: (A) a multimedia article with audio recording on how to deal with the angry behaviors of persons with dementia; (B) a short video clip as a case study to explain angry behaviors of persons with dementia; and (C) push notifications and progress summary.

WECARE is a 7-week program with each week focused on a theme and the final week for summary and additional resources. These themes include (1) facts of ADRD and caregiving; (2) enhancement of caregiving skills; (3) effective communication with providers, care partners, and family members; (4) problem-solving skills for caregiving stress management; (5) stress reduction and depression prevention; (6) practice of self-care and health behaviors; and (7) social support and available resources. The 7 major themes were derived from the evidence-based programs (see Step 2). The curriculum schedule is detailed in Multimedia Appendix 1. The sample screenshots of WECARE are illustrated in Figure 1. By subscribing to the official WECARE account on their own WeChat accounts, participants could receive interactive multimedia programs on their smartphone or tablet once a day, 6 times a week, for 7 weeks. Participants who did not open the program components within a week would be reminded to do.

### Cultural Adaptation

The content of the WECARE program was adapted for Chinese American culture based on input from our stakeholders, and the following changes were made. First, we ensured that the language translation reflected Chinese values and tradition. For example, following the tradition of respecting seniority, Chinese participants did not like the literal translation of “care recipient” or “care partner”; instead, they preferred to use “the elder” (“Lao Ren” in Chinese) to refer to the care partner, which we adopted in the WECARE program. Second, program components specific for our target users were added. For example, given that many Chinese caregivers have limited English proficiency, we added a section on how to communicate with health care providers with a practical checklist as well as a list of terminologies commonly used in medical encounters and dementia care. Additionally, as many Chinese caregivers have limited knowledge and use of formal care and support services [22], we added a section on local resources specific to the participant’s location, including health insurance, dementia care support, transportation assistance for medical care, and translation services. Third, we modified components to meet users’ demand and characteristics. For example, many stakeholders demanded “useful skill-building” content; we included videos demonstrating caregiving skills such as how to transfer, bath, feed, and clean the care partner. We also included many real-life cases to illustrate how to deal with difficult situations such as when a care partner has problematic behaviors in the public. Fourth, we adapted components to reflect cultural practice. For example, many Chinese caregivers live in multigenerational households and value “filial piety”; thus, we modified the component on how to communicate between family members, including communication on how to share responsibilities and how to discuss sensitive topics such as death. Additionally, as stress and depression are not commonly discussed in Chinese culture, we included a section to explain the importance of stress reduction and depression prevention from the perspective of family and love, citing real-life stories and demonstrating practical stress-reduction techniques.

### Multimedia Features

The WECARE program consists of multimedia components to engage users and enhance understanding. Considering that many caregivers are older adults and many have lower levels of health literacy, each article is accompanied by an audio recording, so participants with vision impairment can listen to audio recordings for most of the WECARE content. We also included many short videos (3-5 minutes) adapted from other caregiver interventions with subtitles for illustration. Culturally relevant characters, storylines, and background music are embedded in all program components.

### Social Networking and Social Support

As WECARE is delivered via the popular WeChat social media app, the built-in functions of social networking in WeChat were used to enhance social support among participants. For example, participants were invited to attend staff-moderated group meetings scheduled at week 3, week 5, and week 7 and welcome to share their personal experiences during the course of WECARE. Participants could also initiate their own “group chat”; in a group chat, they could “friend” any fellow caregiver participant for a private chat. Prior to the group meetings, participants agreed to the protocol that all participants in the group meetings address each other by first names only and that no personal information discussed in the meetings be shared with other people outside the group.

### Back-end Database

**Figure 2:**
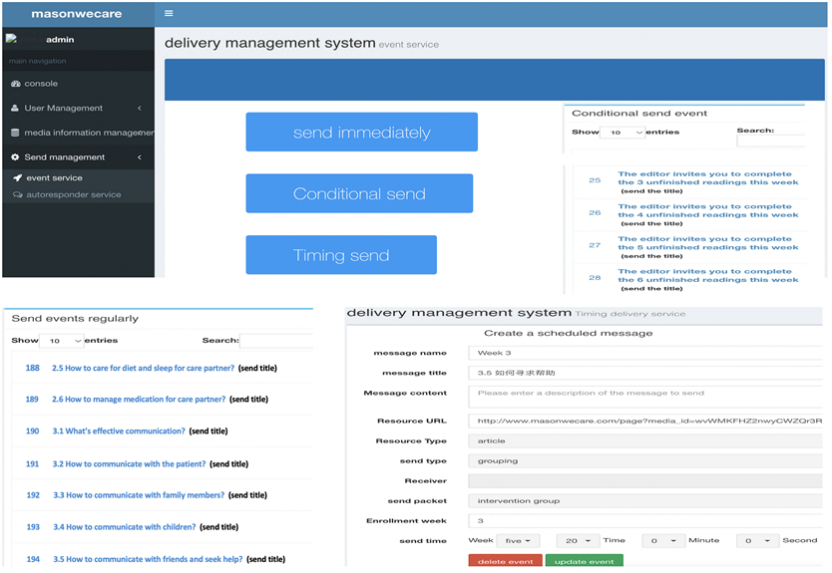
Back-end database: scheduled delivery system.

**Figure 3:**
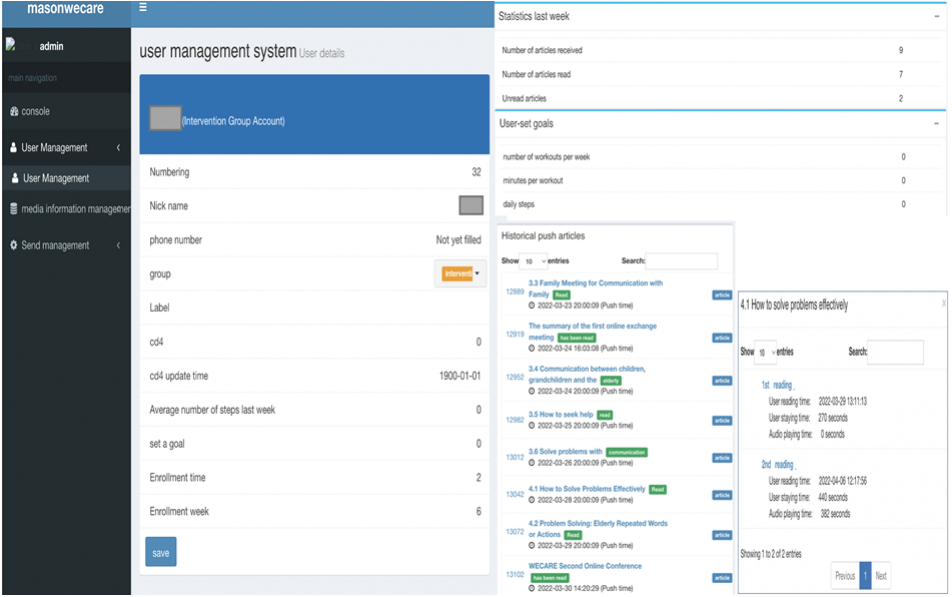
Back-end database: user management system.

Along with developing the front-end program components of WECARE, we also created an interactive web-based application to serve as the back-end database to manage intervention delivery and monitor user engagement. This interactive website has 3 core functions: (1) storing information on every participant enrolled, including characteristic and user preferences; (2) pushing the program components based on user preferences and response (eg, if a participant does not open the WECARE program component within a week, a reminder message will be sent); and (3) tracking program receipt and responses. User engagement indicators tracked in the database include whether a program component is opened, how many times the program is opened or played, and how much time is spent on each component. Figure 2 illustrates the scheduled delivery system through which the components of the WECARE program can be sent to users at prescheduled time. Figure 3 illustrates the user management system that stores user information and tracks user activities.

## Discussion

### Principal Findings

This study reports the process of developing WECARE, a culturally tailored social media–based intervention to enhance caregiving skills and reduce psychosocial distress in Chinese American caregivers of persons with dementia, and its key features. Community-based user-centered design principles were applied in the intervention development. We first crafted the structure of the WECARE curriculum based on existing evidence-based interventions and input from experts. Second, working closely with key stakeholders and through an iterative design-discuss-revise process, we developed culturally tailored multimedia program components at the frontend and an interactive database for intervention delivery and user profile management at the backend. Finally, we conducted a beta test of the complete WECARE program in target users and further refined it. The resulting WECARE is a 7-week mHealth program. Through subscribing to the official WECARE account, users could receive 6 multimedia articles pushed to their WeChat account each week for 7 weeks. Users could also use the built-in functions of WeChat for social networking. The back-end database automatically pushes program components with a preset schedule and tracks user activities on WECARE.

### Strengths

#### The First mHealth Intervention for Chinese American Caregivers of Persons With Dementia

The WECARE program represents one of the first culturally tailored mHealth programs for Chinese American caregivers of persons with dementia. The health disparities experienced by minority and immigrant caregivers of persons with dementia have been exacerbated during the COVID-19 pandemic. There is an urgent need to adapt evidence-based interventions for wider dissemination in underserved populations. The cultural adaptation and digitalization of evidence-based interventions, such as developing the WECARE program as described in this paper, could be an effective approach to address the literature gap and public health needs.

#### Capitalizing on a Popular Social Media App for Intervention Delivery

When adapting and developing mHealth interventions, we need to consider the mobile use behaviors of the target population. The popular social media apps used by minority populations can serve as an efficient channel for intervention delivery [37]. The WECARE program is delivered via WeChat, a social media app with a high penetration rate in Chinese Americans. This program is the first time a social media app popularly used by minority populations is being used to deliver an intervention for minority caregivers of persons with dementia. Delivering mHealth interventions via such apps can save costs, increase accessibility, and enhance sustainability [38].

#### Cultural Adaptation for Target Users

Cultural sensitivity is critical for developing interventions for minority populations. A community-engaged user-centered design is an effective approach for the cultural adaptation of evidence-based programs. Using this approach, we made substantial changes in the WECARE program components in response to the input of our stakeholders and target users. The resulting program reflects the values, needs, and practices of Chinese American caregivers of persons with dementia.

#### Multimedia Features

The WECARE program features multimedia components of audio recording, short video clips, pictorial messages as well as automatic and interactive delivery based on user preferences and responses. The built-in functions in WeChat such as “private chat,” “group chat,” and “video chats” facilitate social networking and enhance social support. Literature suggests that mHealth programs with multimedia features and social networking functions are likely to engage target users, especially those with lower levels of health literacy [39]. The design and innovative features of WECARE will inform future designs of mHealth interventions for caregivers.

#### Back-end Database

The back-end database has the functions of user profile management, the automatic delivery of program components, and user activity tracking. These functions are similar to another WeChat-based intervention and enable potential scale-up and long-term follow-up [40]. The user activity tracking function also allows a future study to examine the relationship between user engagement and intervention effect [41].

### Limitations

First, the beta test of the WECARE program was based on a small sample of 5 target users. The development was a rigorous process following community-engaged user-centered design principles [36] with input from experts and iterative discussion with key stakeholder. Further, according to Nielsen [42], 5 users are sufficient to elaborate usability. Second, the back-end database was designed specifically for WECARE delivery and requires a software engineer. We are seeking open-sourced solutions to lower costs and increase the potential of WECARE’s adoption and scale-up. Third, the biggest limitation of the study is that we do not have data on the feasibility and effectiveness of WECARE as a complete program as well as user feedback on its innovative features and functions. A pilot study is ongoing, and the results will be available once the trial is complete.

### Future Directions

In conclusion, this paper reports the development process and key features of the WECARE program—a culturally tailored, linguistically appropriate, and interactive social media–based interventions to improve caregiving skills and reduce psychosocial distress among Chinese American caregivers of persons with dementia. As the US population becomes older and more diverse, an urgent need exists for more culturally sensitive mHealth interventions for minority and immigrant caregivers of persons with dementia. We advocate for more research and practice of the cultural adaptation of evidence-based program for digital delivery, capitalizing on the widespread use of smartphones and highly popular social media apps, to meet the needs of racial/ethnic minority and immigrant patients with dementia and their caregivers.

## Appendix

Multimedia Appendix 1 Wellness Enhancement for Caregivers (WECARE) program schedule.

## Abbreviations

ADRD: Alzheimer disease or related dementias
mHealth: mobile health
WECARE: Wellness Enhancement for Caregivers
