# Supplementary material for: A Social Media–Based Intervention for Chinese American Caregivers of Persons With Dementia: Protocol Development
Source: JMIR Aging. 2022 Sep 29;5(3):e40171. doi: 10.2196/40171 (PMC9562087; doi:10.2196/40171)
Supplement: Multimedia Appendix 1 [file aging_v5i3e40171_app1.pdf]

Multimedia Appendix 1

Table S1: WECARE Program Schedule

**Week 1: Program overview and introduction**	
1.1	Introduction of WECARE
1.2	What is AD? What's dementia? What are the symptoms, causes, and treatment
1.3	How to provide care for AD patients in different stages?
1.4	How to seek medical help as a CP or caregiver?
1.5	The roles of caregiver
1.6	Week 1 summary: What's ADRD and what does it mean to be a caregiver?
**Week 2: Caring for ADRD patients**	
2.1	How to transfer CP and prevent injuries?
2.2	How to care for CP's oral health?
2.3	How to help CP bath and dress?
2.4	How to help CP use toilet?
2.5	How to care for diet and sleep for CP?
2.6	How to manage medication for CP?
**First group meeting**	
**Week 3: Effective communication**	
3.1	What's effective communication?
3.2	How to communicate with the patient?
3.3	How to communicate with family members?
3.4	How to communicate with children
3.5	How to communicate with friends and seek help?
3.6	Week 3 Summary: Communication as a strategy to minimize conflict, identify a solution, and collaborate for a solution.
**Week 4: Problem solving in caregiving**	
4.1	What's Problem Solving?
4.2	Problem solving example 1: How to deal with repetitive behaviors of CP?

4.3	Problem solving example 2: How to deal with CP's aggressive behaviors?
4.4	Problem solving example 3: When other family members don't take their share of responsibility for CP
4.5	Problem solving example 4: CP doesn't want to take medicine
4.6	Week 4 summary: General rules in dealing with difficult behaviors
**Second group meeting**	
**Week 5: Stress reduction and depression prevention**	
5.1	What's stress? What are the signs you are stressed (emotional & physical)?
5.2	How to deal with stress?
5.3	How to deal with negative emotion?
5.4	How to prevent depression?
5.5	Mindfulness as an approach to manage stress and negative emotions
5.6	Week 5 summary: Manage your emotional health and mental health
**Week 6: Becoming a healthy caregiver**	
6.1	Caregivers' self-care needs
6.2	Nutrition to stay healthy
6.3	Regular exercise to stay healthy
6.4	How to sleep well
6.5	Learn to relax and rest well
6.6	Week 6 summary: Importance of self-care
**Third group meeting**	
**Week 7: Course summary**	
7.1	Summary of how to take care of CP, including difficult behaviors (Weeks 1-2)
7.2	Summary of how to communicate effectively and Problem-Solving mindset and skills (Weeks 3-4)
7.3	Summary of self-care: Caregivers' mental and physical health (Weeks 5-6)
7.4	Plan the future: make plans for every day, making end-of-life decisions
7.5	Week 7 summary and list of local resources

CP=care partner, AD=Alzheimer's disease
